# Reasons for current pregnancy amongst grand multiparous Gambian women - a cross sectional survey

**DOI:** 10.1186/s12884-016-1016-7

**Published:** 2016-08-11

**Authors:** Patrick Idoko, Glenda Nkeng, Matthew Anyawu

**Affiliations:** 1 Edward Francis Small Teaching Hospital, Independence Drive, Banjul, The Gambia; 2 School of Medical and Allied Health Sciences, University of The Gambia, Independence Drive, Banjul, The Gambia

**Keywords:** Grand multiparity, Reasons, Gambia, Contraception

## Abstract

**Background:**

While grand multiparity is now relatively rare in the developed world it is still common in Sub Saharan Africa. Although significant resources have been committed to providing modern contraceptive methods in the Gambia, the total fertility rate is still high at 5.6. Determining the reasons grand multiparous women proffer for the current pregnancy may help in understanding this trend and tailoring appropriate messages to address any specific concerns.

**Method:**

A cross sectional survey of grand multiparous women was carried out at the Royal Victoria Teaching Hospital (now Edward Francis Small Teaching Hospital) to determine the reasons for the current pregnancy.

**Results:**

The prevalence of grand multiparity was 26.5 % while the average parity among the study population was 7.2 (sd 1.8). The most common reasons given for the current pregnancy were: the desire for another child (22.8 %), the pregnancy was unplanned - a “mistake” (18.4 %) and the need to replace a dead child (15.4 %).

**Conclusion:**

Grand multiparity is still very common in The Gambia. Additional efforts are required to target those with unplanned pregnancies. Improving child survival may also decrease the prevalence of grand multiparity.

**Electronic supplementary material:**

The online version of this article (doi:10.1186/s12884-016-1016-7) contains supplementary material, which is available to authorized users.

## Background

A grand multiparous woman is one who has carried five or more pregnancies to the age of viability [1]. Although grand multiparity does not necessarily end in adverse pregnancy outcomes, studies in Sub-Saharan Africa show that it remains a significant contributor to maternal and perinatal morbidity and mortality [2–4]. It is associated with maternal anaemia in pregnancy, antepartum haemorrhage, abnormal foetal presentation, postpartum haemorrhage as well as medical conditions such as hypertension in pregnancy [2–4]. The grand multiparous woman is also more likely to require a surgical obstetric intervention with its attendant risks [5]. In addition, there are associated perinatal problems including low birth weight, preterm birth and congenital malformations [7].

The risks associated with grand multiparity have been significantly reduced in developed countries since Bethel Solomons first described the phenomenon as the “dangerous multipara” in 1934 [1, 6]. Current incidence in these settings now ranges from 3 to 4 % [8]. This is due to the high literacy level, availability of modern contraceptive methods, liberal abortion laws and the improved health care services which ensure the survival of almost all children [9, 10]. High parity is however still a common problem in obstetric practice in many developing countries with incidence ranging from 17 to 33 % in Sub-Saharan African countries [5, 11, 12]. Thus, grand multiparity could be seen as an indicator of low literacy, poverty and other forms of injustice and inequity faced by women in the developing world. Programs aimed at empowering women such as girl child education take a while to drastically create a significant dent in the grim maternal mortality statistics from Africa. Thus, improving access to effective family planning methods remains one of the cardinal “quick fix” strategies available in the fight to reduce maternal mortality.

The Gambian National Reproductive Health Policy provides for the provision of free family planning services in all the health centres in the country. Despite this huge investment in family planning by the government and international donors, grand multiparity remains a common feature of obstetric practice in The Gambia. The crude birth rate is 27/1000 population while the total fertility rate is 5.6 births per woman [13]. According to WHO estimates, the maternal mortality rate in The Gambia is 430/100,000 live births [14]. The commonest causes of these maternal deaths in the hospital are obstetric haemorrhage, hypertensive diseases in pregnancy and sepsis [15, 16].

Studies from other parts of Sub-Saharan Africa have alluded to various reasons for the current pregnancy amongst grand multiparous women. Prominent amongst these are: the desire for large family size, the desire to have a child for a new husband, gender preferences, loss of a child and failed contraception [17, 18]. This study aimed to describe the reasons for the current pregnancy amongst grandmultiparous women at the antenatal clinic of the Royal Victoria Teaching Hospital (RVTH). The study also assessed the contraceptive knowledge and beliefs about contraception of grand multiparous women.

## Method

The study was conducted at the RVTH (now Edward Francis Small Teaching Hospital), Banjul, The Gambia. RVTH is the only tertiary health facility in The Gambia serving its 1.7 million population. The country is a narrow strip of land bordered on three sides by Senegal with a strip of coastline bordering the Atlantic Ocean. RVTH offers primary health care services to women in the greater Banjul area and also serves as the main referral centre for specialized tertiary maternity and reproductive health services.

A structured, pre-tested interviewer administered questionnaire was used (see Additional file 1: Questionnaire: reasons for current pregnancy amongst grand multiparous Gambian women). The questionnaire was verbally translated into the local languages for those illiterate in English. Ethical approval for the study was obtained from the Royal Victoria Teaching Hospital Ethics Committee before commencement of the study. Written, signed informed consent was obtained from each participant prior to the first study procedure. Information was read to the non-literate and translated verbally into their local language of choice. These individuals were then required to thumb print the consent form.

## Results

A total of 136 grand multiparous women were seen during the study period from a total of 514 antenatal bookings (prevalence of grand multiparity −26.5 %). The mean age of the study population was 35.5 years (sd 4.3). Table 1 details the demographic characteristics of the study population.

**Table 1:** Demographic characteristics of study participants

Age group	Frequency	Percent
> 20–25	3	2.2 %
> 25–30	14	10.4 %
> 30–35	55	40.4 %
> 35–40	48	35.3 %
> 40–45	15	11.0 %
> 45	1	0.7 %
Total	136	100.0 %
Mean Parity	7.2 (sd 1.8)	
Average number of children alive	5 (sd 2.1)	
Education	Frequency	Percent
None	42	30.9
Arabic School only	34	25.0
Primary School	27	19.9
Middle School	26	19.1
High School	7	5.1
Total	136	100.0
Occupation	Frequency	Percent
Petty trader	56	41.2
Cashier	2	1.5
Civil Servant	1	0.7
Farmer	17	12.5
Housewife	46	33.8
Manual Worker	7	5.2
None	1	0.7
Nurse	2	1.5
Teacher	4	2.9
Total	136	100.0
Residence	Frequency	Percent
Rural	40	29.4
Urban	96	70.6
Total	136	100.0
Place of delivery in last pregnancy	Frequency	Percent
Home	9	6.6
Hospital	127	93.4
Total	136	100.0
Antenatal clinic attendance in last pregnancy		
Yes	135	99.3
No	1	.7
Total	136	100.0
Contraceptive counseling in last pregnancy	Frequency	Percent
No	59	43.4
Yes	77	56.6
Total	136	100.0

**Fig. 1:**
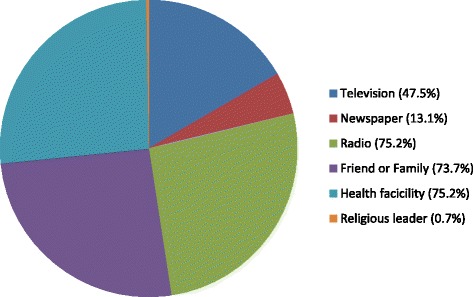
Source of contraceptive knowledge amongst study participants

One hundred and thirty two (97.1 %) reported having previously heard of family planning. Common sources of knowledge of family planning were the health facility and radio (Fig. 1).

Common stories related to contraception that these women had encountered are presented in Table 2, while Table 3 shows the beliefs of participants regarding contraception.

**Table 2:** Common stories about contraception that the participants have heard

	Frequency	Percentage
It causes menstrual abnormalities	15	11.0
It causes severe diseases including cancer	33	24.3
It is effective in child spacing	49	36.0
It decreases the potential for subsequent fertility	20	14.7
It is unreliable (fails easily)	8	5.9
It is forbidden on religious grounds	6	4.4
It generally improves health	5	3.7

**Table 3:** Beliefs about contraception by participants

	Strongly agree	Agree	undecided	Disagree	Strongly disagree
Contraception is against my religious belief	26 (19.1 %)	36 (26.5 %)	36 (26.5 %)	18 (13.2 %)	20 (14.7 %)
Contraceptives can prevent subsequent pregnancy	15 (11.0 %)	28 (20.6 %)	28 (20.6 %)	22 (16.2 %)	43 (31.6 %)
Contraception causes serious illness	21 (15.6 %)	30 (22.2 %)	38 (28.2 %)	24 (17.8 %)	22 (16.3 %)

Less than half (44.9 %) of the study participants had used a modern method of family planning in the past (Table 4). The main reasons given for not using contraception were fear of side effects (33.3 %), refusal of partner (17.3 %) and religious beliefs (16.0 %) (Table 5). Common reasons given for the current pregnancy were a desire for more children, death of another child and “mistake” (Table 6).

**Table 4:** Methods of contraceptives used in the past by study participants

	Frequency	Percentage (%)
Injectable	27	44.3
Pills	27	44.3
IUD	7	11.4

**Table 5:** Reasons given for not using contraceptive

	Frequency	Percentage (%)
Afraid of side effects	25	33.3
Religious beliefs	12	16.0
Did not know about family planning	4	5.4
Did not know where to get	7	9.3
My partner refused	13	17.3
No specific reason	14	18.7
Total	75	100.0

**Table 6:** Reason for current pregnancy in study participants

Reasons for index pregnancy	Frequency	Percent
Death of another child	21	15.4
Failed Contraception	11	8.1
Gender related	11	8.1
Husband’s Wish	5	3.7
Mistake	25	18.4
More Children	31	22.8
No Reason	17	12.5
Others	3	2.2
Remarried	12	8.8
Total	136	100.0

While 95 (69.9 %) of the participants were willing to use a contraceptive method at the end of the current pregnancy, 40 (29.4 %) were not willing to use any method and one person (0.7 %) was undecided.

Seventy-five (55.1 %) said their partner would support their decision regarding family planning while 51 (37.5 %) said partner would not support their decision and 10 (7.4 %) were unsure.

## Discussion

The prevalence of grandmultiparity in our study was 26.5 %. This is similar to other studies conducted in the sub-region with prevalence ranging from 17 to 33 % [5, 11, 12]. However, in developed countries, grand multiparity is comparatively lower ranging from 3 to 4 % [8]. This difference has been attributed to disparity in literacy level, health care services, availability, accessibility of modern contraceptive methods and differing abortion laws [9, 10].

Evidence of adverse pregnancy outcome in high parity is overwhelming and the risk is higher for great grand multiparous women with over ten children compared to grand multiparous women [4, 19–22]. These obstetric complications include hypertension, gestational diabetes, abruptio placenta, placenta praevia, foetal macrosomia, multiple pregnancy, labour dystocia, uterine rupture and perinatal death [4, 19–21]. Grand multiparity and great grand multiparity are independent risk factors for labour dystocia and perinatal mortality [19].

In our study, the majority (55.9 %) had no formal education and only 6.6 % had a civil job. The majority were housewives (33.8 %) and petty traders (41.2 %). These findings correlate with previous studies that had implicated poverty; poor education and ignorance as the driving force for low uptake of contraceptives and “mistake” as a reason for index pregnancy as seen in this study [17].

**Fig. 2:**
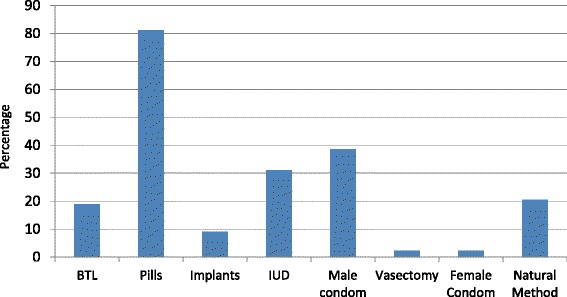
Methods of family planning identified by study participants

The most common reasons given for the current pregnancy were a desire for more children, death of another child or a “mistake” (Table 6). This finding is similar to a Nigerian study that found that women were willing to get pregnant again if their child died or to have a large family [17]. Traditionally, The Gambia is largely an agrarian society that practices subsistence farming. Therefore, a high premium is placed on having a large family size to help with the farm work. Women who said the current pregnancy was a “mistake” are likely implying that they were not ready to get pregnant at the time they got pregnant. This might be highlighting an unmet need for contraception. Knowledge of contraceptive methods (Fig. 2) was however quite high (80 % were aware of pills) but more than 30 % were worried about the side effects, which prevented them from using contraceptives. It is also pertinent to note that more than 45 % believed that contraceptive use was against their religious dogma and 37 % thought that the use of contraceptives would lead to serious illness. The Gambia is predominantly a religious country and religious leaders are as a result very important opinion leaders. Effective communication and advocacy is necessary to get women to practice contraception. Involving religious leaders in this process will likely be key in ensuring uptake, as has been the case for other interventions such as uptake of oral polio vaccine [23]. Given the patriarchal nature of Gambian society, men will also need to be targeted in effective family planning sensitization campaigns as almost 20 % of women were not using contraceptives because their partners did not give them permission.

Our data suggests little awareness of male and female sterilization in study participants. As over 90 % of the participants had their last delivery at a health facility, a case could be made for offering post partum tubal ligation to women who may desire a permanent method of contraception. Postpartum tubal ligation is known to be a safe, cost effective method of contraception [24]. Currently, this is not the policy or practice in the public health institutions in the Gambia.

We demonstrated that 97.1 % of our study participants were aware of the availability of contraception. In fact, 56.6 % of these mothers had been counseled regarding availability, accessibility and various options available for contraception in the hospital before discharge in their previous pregnancy. Majority of them had their last delivery in the hospital (93.4 %) and lived in an urban area (70.6 %). Therefore, inability to use contraception to prevent the occurrence of index pregnancy was not due to lack of contraception or accessibility, rather the beliefs and stories the women heard regarding contraception likely affected willingness to use contraception resulting in the index pregnancy. A study conducted by Adanu et al in the sub-region showed that education influences the uptake of modern contraception among women in Sub-Saharan Africa [25]. This is in keeping with our finding as more than 55 % of our study participants did not have any formal education.

While 95 (69.9 %) of the participants were willing to use a contraceptive method at the end of the current pregnancy, 40 (29.4 %) were not willing to use any method. This is a known precursor of another pregnancy.

Seventy-five women (55.1 %) reported that their partner would support their decision concerning family planning while 51 (37.5 %) felt he would not give support. This is a very important factor as a previous study had shown that strong husband wish is a determinant factor in acceptance and consistent use of contraception [16].

The use of hospital clients as study participants may limit the generalizability of the study. However, it is more likely that those who do not utilize hospital services are more likely to have an unfavourable attitude towards contraception. However, in the Gambian context, more than 90 % of all pregnant women have antenatal care [13]. Therefore, a community-based study may not differ much.

## Conclusion

In conclusion, grand multiparity is still quite common in The Gambia in spite of the availability of modern contraceptive methods. The most common reason given for the current pregnancy among the grand multiparous was a desire for more children. This is closely followed by those who got pregnant by “mistake”. Women need to be informed of the dangers of grand multiparity and advised to practice effective family planning methods to prevent further pregnancy. However, it is imperative to reiterate the importance of quality antenatal care that effectively incorporates the concept of birth preparedness and complication readiness as the effects of these complications can be minimized by good antenatal care. There is however a need to develop family planning messages that specifically target men and religious leaders as they are key opinion leaders and decision makers. Disseminating accurate information on contraceptives to address popular myths on contraception will also be key.. Postpartum contraceptive methods like tubal ligation and the intrauterine contraceptive device should be made readily available and the Gambian reproductive health policy should focus on these methods of contraception in addition to other commonly available contraceptive methods. In the long term, improving access to education for girls will likely reduce the prevalence of grand multiparity.

## Abbreviations

RVTH, Royal Victoria Teaching Hospital

## Additional file

Additional file 1: Questionnaire reasons for current pregnancy amongst grand multiparous Gambian women. (DOC 29 kb)
