# Supplementary material for: Reasons for current pregnancy amongst grand multiparous Gambian women - a cross sectional survey
Source: BMC Pregnancy Childbirth. 2016 Aug 11;16:217. doi: 10.1186/s12884-016-1016-7 (PMC4982305; doi:10.1186/s12884-016-1016-7)
Supplement: Additional file 1: — Questionnaire reasons for current pregnancy amongst grand multiparous Gambian women. (DOC 29 kb) [file 12884_2016_1016_MOESM1_ESM.doc]

**REASONS FOR CURRENT PREGNACY IN GRANDMULTIPAROUS WOMEN AT RVTH ANTENATAL CLINIC**

1. Usual place of residence:
2. Age ( in years):
3. Education: None [ ] Primary [ ] Middle [ ] High [ ] University [ ] Arabic [ ] Others: (specify)________________
4. Religion: Islam [ ] Christian [ ] Others: (specify)____________
5. Ethnic group: Wolof [ ] Mandinka [ ] Fula [ ] Manjago [ ] Sarahule [ ] Aku [ ] Others: Specify________________
6. Occupation:
7. Age at time of first marriage:
8. Number of times you have been married:
9. Length of married life:
10. Number of pregnancies:
11. Number of children alive:
12. Where was your last child delivered: Public health centre /hospital [ ] private clinic/NGO [ ] home[ ] others: Specify
13. Did you attend antenatal clinic during your last pregnancy? Yes [ ] no [ ]
14. Did you receive family planning counseling during the antenatal or postpartum period in your last pregnancy? Yes [ ] no [ ]
15. Reasons for current pregnancy: More children [ ] Children’s death [ ] Mistake [ ]

Gender [ ] Remarry [ ] Failed contraception [ ] Husband’s wish [ ] No reason [ ]

Others: Specify

Part B: KNOWLEDGE, ATTITUDE AND PRACTICE OF FAMILY PLANNING

1. Have you ever heard about family planning? Yes [ ] no [ ]
2. What is your source of information on family planning? Newspaper [ ] radio [ ] television[ ] friend or family [ ] health worker or health facility [ ] religious leader [ ] partner [ ] others [ ] specify ---
3. Have you ever been counseled on family planning during any of your previous pregnancies in the antenatal or postnatal clinics? Yes [ ] no [ ]
4. List all the modern methods of family planning you know. ----------------------------------------------------------------------------------------------------------------------------
5. What are some of the common stories about family planning that you have heard? ------------------------------------------------------------------------------------------------------------------------------------
6. Family planning is against my religious beliefs. Strongly agree [ ] agree [ ] undecided [ ] disagree [ ] strongly disagree [ ]
7. Family planning methods prevents women from getting pregnant subsequently. Strongly agree [ ] agree[ ] undecided [ ] disagree [ ] strongly disagree[ ]
8. Family planning methods can cause a woman to have serious illness including cancer or death. Strongly agree [ ] agree [ ] undecided [ ] disagree [ ] strongly disagree [ ]
9. Have you ever used any form of family planning in the past? Yes [ ] no [ ]
10. If yes, what methods did you use & how long for each method? --------------------------------------------------------------------------------------------------------------------------------------------------------
11. If yes, why did you stop using the family planning method? Side effects [ ] my partner wanted me to stop [ ] I wanted to get pregnant [ ] no reason [ ] others [ ] specify -
12. If you have never used any method of family planning before why? I did not know about family planning [ ] I did not know where to obtain family planning methods from[ ] it was too expensive[ ] my partner refused [ ] against my religious belief [ ] afraid of side effects [ ] others [ ] specify--------
13. Are you willing to use a family planning method after this current pregnancy? Yes [ ] no [ ]
14. Is your partner involved in taking decisions on family planning? Yes [ ] no [ ]
15. Will your partner actively support your decision on family planning? Yes [ ] no [ ] I don’t know [ ]
16. What is the average age gap between your children? ------years

Name of interviewer:__________________________
